# Kidney volume to GFR ratio predicts functional improvement after revascularization in atheromatous renal artery stenosis

**DOI:** 10.1371/journal.pone.0177178

**Published:** 2017-06-08

**Authors:** Constantina Chrysochou, Darren Green, James Ritchie, David L. Buckley, Philip A. Kalra

**Affiliations:** 1 The University of Manchester, Manchester Academic Health Science Centre, Salford Royal NHS Foundation Trust, Stott Lane, Salford, United Kingdom; 2 Division of Biomedical Imaging, University of Leeds, LIGHT Laboratories, Leeds, United Kingdom; Universidade Estadual Paulista Julio de Mesquita Filho, BRAZIL

## Abstract

**Background:**

Randomized controlled trials (RCT) have shown no overall benefit of renal revascularization in atherosclerotic renovascular disease (ARVD). However, 25% of patients demonstrate improvement in renal function. We used the ratio of magnetic resonance parenchymal volume (PV) to isotopic single kidney glomerular filtration rate (isoSKGFR) ratio as our method to prospectively identify "improvers" before revascularization

**Methods:**

Patients with renal artery stenosis who were due revascularization were recruited alongside non-ARVD hypertensive CKD controls. Using the controls, 95% CI were calculated for expected PV:isoSK-GFR at given renal volumes. For ARVD patients, “improvers” were defined as having both >15% and >1ml/min increase in isoSK-GFR at 4 months after revascularization. Sensitivity and specificity of PV:isoSK-GFR for predicting improvers was calculated.

**Results:**

30 patients (mean age 68 ±8 years), underwent revascularization, of whom 10 patients had intervention for bilateral RAS. Stented kidneys which manifested >15% improvement in function had larger PV:isoSK-GFR compared to controls (19±16 vs. 6±4ml/ml/min, p = 0.002). The sensitivity and specificity of this equation in predicting a positive renal functional outcome were 64% and 88% respectively. Use of PV:isoSK-GFR increased prediction of functional improvement (area under curve 0.93). Of note, non-RAS contralateral kidneys which improved (n = 5) also demonstrated larger PV:isoSK-GFR (15.2±16.2 ml/ml/min, p = 0.006).

**Conclusion:**

This study offers early indicators that the ratio of PV:isoSK-GFR may help identify patients with kidneys suitable for renal revascularization which could improve patient selection for a procedure associated with risks. Calculation of the PV:isoSK-GFR ratio is easy, does not require MRI contrast agent.

## Introduction

Atherosclerotic renovascular disease (ARVD) is highly prevalent amongst patients with CKD (up to 15% of CKD diagnoses [1]), and also those with atheromatous disease in other organ systems (e.g. 47% of patients undergoing coronary artery bypass grafting have ARVD [2]). Survival is worse amongst ARVD patients than other causes of CKD [3]. Two large clinical trials have shown no worthwhile benefit of renal artery revascularization as first line therapy over medical therapy alone for renal artery stenosis (RAS) [4,5].

However, many of these patients had asymptomatic or incidental renovascular disease, and others were excluded on the basis of an apparent pre-existing indication for intervention. Questions therefore remain regarding the wider applicability of the results of ASTRAL and CORAL to all patients with ARVD [6].

It is recognised that many individuals with RAS do benefit from stenting. Observational studies demonstrate that 20–30% of patients show improvement in renal function after revascularization [7,8]. In addition, 7% of ASTRAL patients randomized to revascularization experienced a complication from the procedure. What is not clear is how to predict which patients will respond favourably. This would assist in reducing inappropriate and potentially harmful interventions.

The diversity in renal functional outcome after revascularization is thought to reflect the spectrum of the relative contributions of pre-existing parenchymal damage from long standing hypertensive, atheromatous and cytokine insult, in addition to the simple hemodynamic effects of the arterial narrowing, in the pathogenesis of the renal dysfunction [9, 10]. In most patients, ARVD is a chronic process with a significant duration of hypertensive stress and renal remodelling as a response to cytokine release [11]. Kidneys which demonstrate functional improvement following revascularization may reflect a subgroup which have not undergone irreversible parenchymal structural damage. In a magnetic resonance imaging (MRI) study by Cheung et al, we demonstrated the characteristics of a sub-group of kidneys that manifest improved renal function post renal revascularization. These kidneys displayed a larger MRI assessed parenchymal volume (PV) to radioisotope assessed single kidney glomerular filtration (isoSK-GFR) [12]. The term ‘hibernating parenchyma’ can be used to describe this subgroup of kidneys where renal dysfunction is thought to be potentially reversible with treatment of the stenosis. This methodology was non–invasive and, although it used MRI, this did not require administration of a gadolinium-based contrast agent. Also, this and most other studies have not evaluated whether renal functional improvement after revascularization translates into a survival benefit.

The aims of this study were: 1) to prospectively assess the validity and predictive power of the PV:isoSK-GFR measurement; 2) to observe the longterm outcome in patients predicted to improve after revascularization compared to those patients predicted to not improve. We also studied the PV: isoSK-GFR relationship in non-RAS contralateral kidneys to determine whether revascularization of a stenosed kidney can incidentally result in improvement in non-RAS contralateral kidneys as a result of change in systemic neurohumoral responses [13].

## Materials and methods

### Patient recruitment

Ethics approval was granted by South Manchester Ethics Committee UK REC ref: 07/Q1405/21 in accordance with the Declaration of Helsinki, and all patients gave written informed consent prior to inclusion. Patients presenting to our tertiary referral centre between January 2007 and December 2010 who were due to undergo renal revascularization were approached to participate in this study. The clinical decision to consider revascularization was based on: 1) clinical grounds (e.g. poorly controlled hypertension on 4 or more anti-hypertensive agents, deteriorating renal function, or flash pulmonary oedema) or 2) randomization into the revascularization arm of an ongoing clinical trial, either the ASTRAL (ISRCTN59586944) [4] or CORAL[5] (NCT0008173) trials.

Control patients were those attending the renal clinic in whom there had been clinical suspicion of ARVD (i.e. signs and symptoms suggestive of renovascular disease such as poorly controlled hypertension, CKD, arterial bruits or discrepant kidney sizes), but who had no evidence of RAS detectable by MR or CT angiography. The purpose of this control group was to assess renal PV:isoSK-GFR changes in hypertensive patients without RAS (rather than in healthy controls) in order to devise an appropriate reference group for comparison of PV:isoSK-GFR.

### Sample size calculation

From our pilot study [12], kidneys that showed a difference between actual and predicted PV:isoSK−GFR of >2ml/ml/min were more likely to show improved renal function after revascularization compared to those with PV:isoSK−GFR differing by <2ml/ml/min. Using the SD, which was 1.5ml/ml/min, the number of kidneys (that manifest improved function) required to detect a difference of 2ml/ml/min between actual and predicted PV:isoSK−GFR with a significance level of 0.05 and 90% power was 15. In the pilot study, 7 of the 16 revascularized kidneys (44%) showed improved function. Hence, it was estimated that 35 kidneys would need to be revascularized within this study.

### Radioisotope single-kidney GFR (isoSK-GFR)

All patients underwent baseline assessment of individual kidney function involving a standard radioisotopic methodology [14–16]. Overall GFR was first measured with ^51^Cr-Ethylenediaminetetraacetic acid (^51^Cr-EDTA) clearance. ^99m^Tc-dimercaptosuccinic acid (^99m^Tc-DMSA) scintigraphy was then used to assess the differential static radioisotope uptake of each kidney. The isoSK-GFR was expressed as an absolute individual value in ml/min and not ml/min/1.73m^2^ as body surface area was calculated for each patient. In patients who underwent renal revascularization, isoSK-GFR was also repeated at 4 months following the procedure. The daily variation in isoSK-GFR has been shown to be 8.8% [14]. The criteria for clinically relevant change in individual renal function were >15% and >1ml/min increase of isoSK-GFR at 4 months post-revascularization compared to baseline. This was to account for small, low GFR kidneys which may experience a large percentage increase which does not translate to a large absolute increase in ml/min. Following revascularization, both the revascularizaed and contralateral kidneys were classified as having improved, remained stable or deteriorated based upon the following thresholds: improved = isoSK-GFR increased by at least 1ml/min and by >15% compared to baseline; deteriorated = isoSK-GFR decreased by at least 1ml/min and by >15% compared to baseline; stable = changes between the above 2 definitions.

### Magnetic resonance imaging

Data were collected using a 3 T whole-body MR scanner (Philips Acheiva, Philips Medical Systems Lts, NL). Following acquisition of scout images a 3D imaging volume was acquired using a T1-weighted spoiled gradient echo acquisition in the oblique-coronal plane. The volume encompassed both kidneys and the descending aorta and the data were obtained in a single breathhold. Gadolinium was not used for acquisition of the data. The following parameters were used for the acquistion: TR = 4.0ms, TE = 1.0ms, FOV = 400x400x80mm, flip angle = 14 degrees, 2 signal averages, parallel acquisition using a SENSE factor = 2, following interpolation in the Fourier domain matrix = 256x256x40). The parenchymal volumes were calculated from the 3D volume images using the voxel-count method as we have previously described[12,17].

$$\text{Renal volume }\left(\text{ml}\right)=\sum {\left(\text{parenchymal area x slice thickness}\right)}_{\text{n}’th\text{ slice}}$$

### Analysis of PV: isoSK-GFR

With renal damage and shrinkage, loss of cortical tissue occurs to a greater degree than that of the medulla [18] and hence the ratio of PV to isoSK-GFR would increase as the kidney decreases in size. Control kidneys were grouped into 5 categories according to their parenchymal volume and these groups used to define reference ranges for PV:isoSK-GFR ratio at a given volume. Kidneys were considered to have a high PV:isoSK-GFR ratio, and therefore be predicted to respond to revascularisation, when this exceeded the upper limit of the 95% CI for a control kidney of a given volume group. These criteria were set pre-emptively, before the study began.

### Evaluation criteria and statistical analysis

Parametric data are presented as mean±standard deviation and non-parametric data as median (interquartile range). Differences in baseline continuous variables between groups were assessed using ANOVA with natural log transformation applied to non-normal varaibles; baseline categorical variables were compared using chi-square test.

To account for possible effects of regression to the mean due to the non-randomized design, between group comparison of MRI parameters of kidneys with stable, improving or deterirorating function post-revascularization was performed using analysis of covariance (ANCOVA) incorporating follow-up isoSK-GFR data in a least squared means model. Homogeneity of slopes was assessed by including a class-by-covariate interaction term and ANCOVA plots (negative in all cases). Standard error results were calculated to compare the study population mean versus the mean of the true population.

As follow up isoSK-GFR data were not obtained for control kidneys, comparison between these and revascularized kidneys was made using standard ANOVA methodology. Sensitivity / specificity analysis was performed using receiver operating characteristic (ROC) curves and integrated discrimination improvement analysis with isoSK-GFR data considered in addition to baseline renal functional parameters.

A survival analysis was performed to compare outcome in improvers versus non-improvers. Because some patients underwent bilateral revascularization, for this analysis responders were defined as those patients with improvement in isoSK-GFR >15% of any revascularized kidney. Outcome was all-cause mortality and follow up was until death or 31^st^ December 2015. A Cox proportional hazard model was used, adjusted for age and overall GFR at baseline. The study was not powered specifically for this survival analysis.

In all cases statistical significance was defined as an alpha value of <0.05. Analyses were performed in SAS version 9.2 (SAS Institute, Cary, NC, USA) under licence to the University of Manchester with figures generated in R version 3.0.1.

## Results

**Fig 1:**
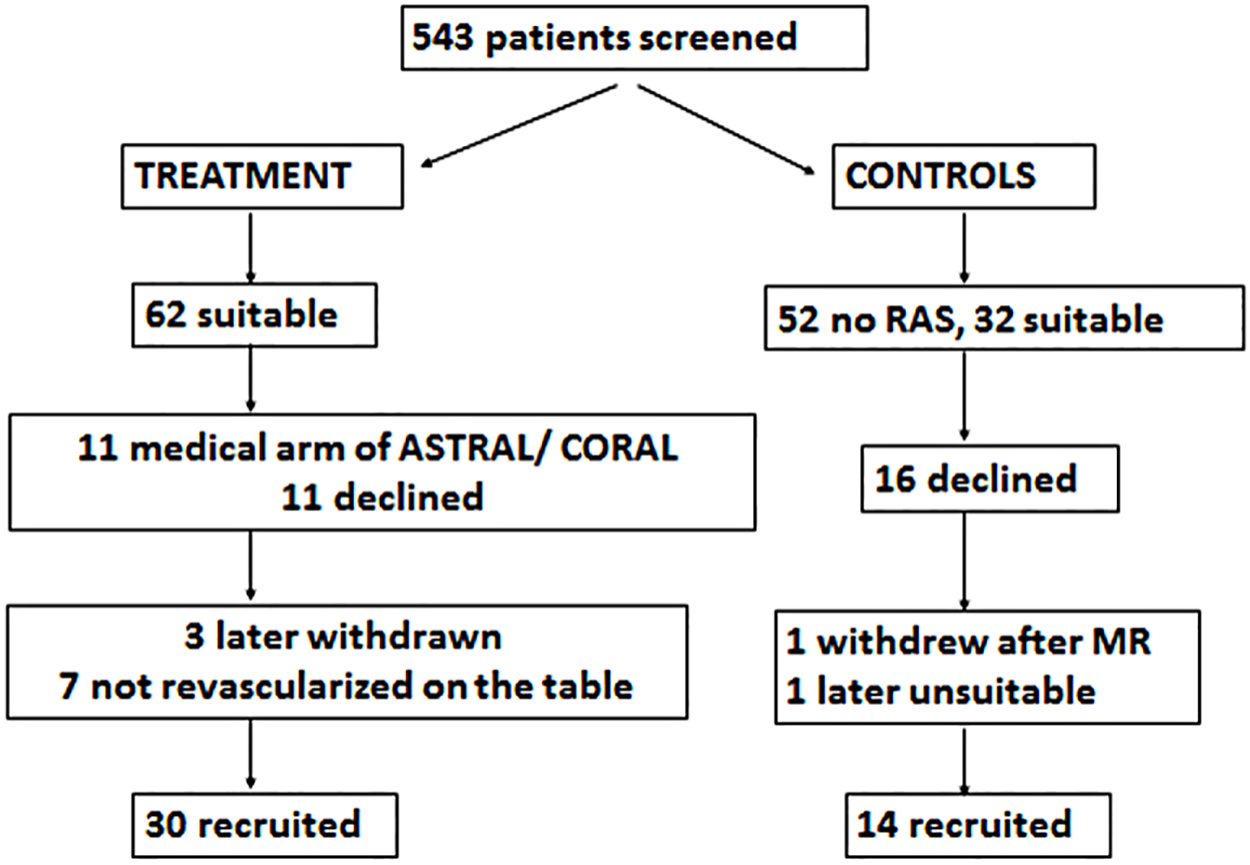
Summary of recruitment flow.

During the study period, 275 patients were screened for ARVD. These patients had a mean age of 68±10 years. Patient recruitment is summarised in Fig 1, including the individual reasons for non-inclusion. There were 30 ARVD patients who underwent revascularization that were included in the final analysis, and 14 non-ARVD hypertensive CKD controls.

The baseline phenotype of the study subjects is shown in Table 1. Revascularized patients were significantly older than non-ARVD controls, had a lower GFR, and were more likely to have smoked. Of the 30 patients who were revascularized, 20 had unilateral and 10 bilateral revascularization. This gave a total of 40 revascularized kidneys for analysis, and 20 contralateral non-RAS kidneys.

**Table 1:** Baseline demographics of control and revascularized subjects.

	Treatment group		Control group		p
	Mean (SD)	Range	Mean (SD)	Range	
**N**	30		14		**-**
**Age (years)**	68.9 (8.9)	51–82	59.4 (12)	39–81	0.005
**Male (n, %)**	19 (63.3%)		7 (50%)		0.402
**Weight (kg)**	75.7 (14.4)	55–110	85 (20.8)	44–115	0.089
**Height (cm)**	164.2 (8.5)	143–178	166.6 (12.2)	150–185	0.46
**BMI (kg/m**^**2**^**)**	28 (4.4)	21–38	31.5 (7.7)	15–47	0.062
**SBP (mmHg)**	158.4 (30.9)	103–214	148.6 (32.5)	107–220	0.339
**DBP (mmHg)**	79.8 (17.9)	37–109	83.1 (12.3)	56–109	0.545
**N anti-hypertensives**	3.7 (2.2)	0–10	2.8 (2)	0–7	0.176
**Biochemical parameters**					
**eGFR (ml/min/1.73m**^**2**^**)**	41.4 (22.6)	12–90	59.8 (19.6)	32–90	0.012
**Isotopic GFR (ml/min)**	35.1 (18.9)	7–72	56.2 (25)	25–112	0.003
**Haemoglobin (g/l)**	123.2 (16.9)	84–164	131.6 (16.9)	105–154	0.145
**uPCR (g/mol)**	63 (121)	0–568	40 (60)	0–158	0.991
**Serum albumin (g/l)**	45.8 (7.3)	40–80	45.1 (2.7)	41–48	0.737
**Cholesterol (mmol/l)**	4.2 (0.9)	3–6	4.4 (0.8)	3–6	0.478
**Comorbid conditions**					
**Hypertension**	29 (96.7%)		13 (92.9%)		0.572
**Previous MI**	12 (40%)		2 (14.3%)		0.088
**PVD**	7 (23.3%)		2 (14.3%)		0.488
**Type II diabetes**	11 (36.7%)		4 (28.6%)		0.598
**Current smoker**	5 (16.7%)		3 (21.4%)		0.703
**Ex-smoker**	13 (43.3%)		1 (7.1%)		0.016
**Prescribed medications**					
**RAAS blockade**	22 (73.3%)		7 (50%)		0.128
**Aspirin**	20 (66.7%)		7 (50%)		0.290
**Statin**	23 (76.7%)		11 (78.6%)		0.888

Estimated GFR calculated using 4-variable MRDR equation. Angiotensin blockade defined as prescription of angiotensin converting enzyme inhibitor, angiotensin II receptor blocker, or direct renin inhibitor.

Indications for revascularization were: randomized to revascularization within the ASTRAL and CORAL trials (n = 7), severe hypertension (n = 8), rapidly deteriorating renal function (n = 9), flash pulmonary oedema (n = 2), poorly controlled heart failure and chest pain (n = 1), to preserve renal mass or prevent renal artery occlusion (RAO) during planned cardiac bypass (n = 2), to enable use of renin angiotensin blockade (RAB) for treatment of hypertension and proteinuria (n = 1).

### Renal functional response in individual kidneys

**Fig 2:**
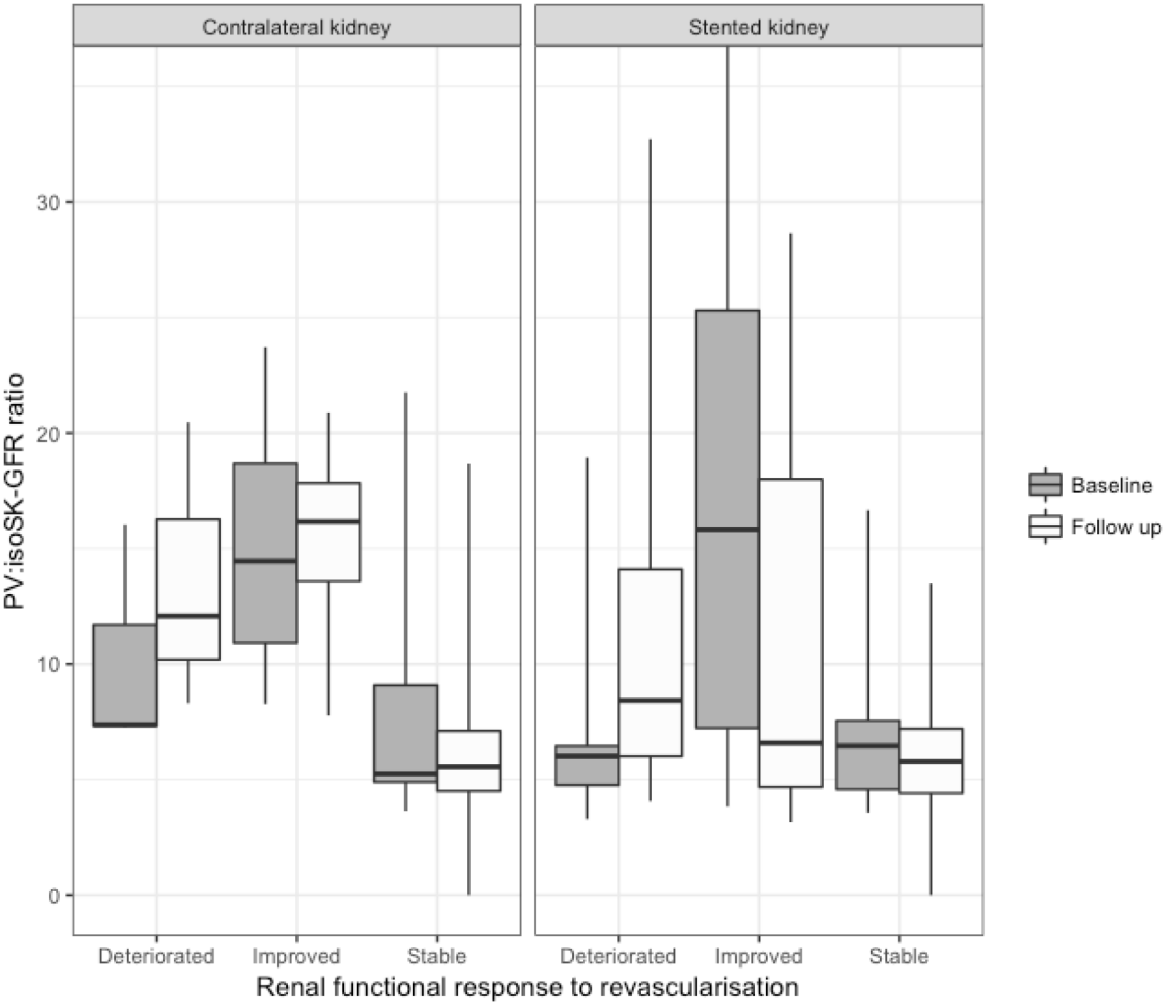
Box plot showing baseline (grey) and follow up (white) PV:isoSK-GFR characteristics in improver, stable and deteriorator kidneys. *P<0.05 within group.

There was no significant difference in baseline renal function between stented kidneys and contralateral kidneys, although contralateral kidneys which remained stable tended to have better baseline isoSK-GFR and larger volumes. Stented kidneys which improved had a mean PV:isoSK-GFR ratio 2.8 times larger than those which remained stable or deteriorated, and 3.1 times greater than controls. The PV:isoSK-GFR ratio of stented kidneys which improved was noted to decrease significantly post revascularization (Fig 2).

Contralateral kidneys that improved had PV:isoSK-GFR ratios 1.7 and 1.5 times greater than contralateral kidneys which remained stable or deteriorated respectively, and 2.4 times greater than control kidneys.

The mean change in overall radioisotope GFR from pre-stent baseline to 4 months post-procedure for the 30 patients who underwent revascularization was 1.3±12.4 mL/min. For the 40 stented kidneys, the mean change in isoSK-GFR was 1.2±10.2 mL/min and for the 20 non-RAS contralateral kidneys the mean change was 0.5±2.1 mL/min. Differences in MRI and radioisotope measurements between patients with improving / stable / deteriorating isoSK-GFR after revascularization are shown in Table 2.

**Table 2:** Comparison of imaging measurements between groups divided by renal functional outcome, comparing these outcome groups for stented ARVD kidneys and non-ARVD contralateral kidneys. Between group statistical analysis is by ANCOVA.

Outcome	Stented kidneys (n = 40)			Contralateral kidneys (n = 20)		
	SK-GFR (mL/min)	PV (mL)	Ratio (min^-1^)	SK-GFR (mL/min)	PV (mL)	Ratio (min^-1^)
Improved >15%	11.0 (2.2)	113.4 (3.9)	19.4 (16.2)	13.7 (0.9)	102.4 (6.2)	15.2 (6.2)
Stable	20.6 (1.6)	124.4 (2.9)	7.0 (3.3)	14.9 (0.6)	106.3 (3.8)	8.7 (6.7)
Deteriorated >15%	25.1 (2.6)	121.9 (4.5)	7.1 (5.0)	16.8 (1)	110.2 (5.7)	10.0 (5.2)
P	<0.001	0.01	<0.001	0.09	0.6	0.23

Results are presented as mean (standard error). GFR = isotope glomerular filtration rate, PV = parenchymal volume, SK = single kidney, Ratio = PV divided by GFR.

There was no significant correlation between severity of RAS and isoSK-GFR (r = 0.094, p = 0.384), PV (r = 0.094, p = 0.384) or PV:isoSK-GFR (r = 0.008, p = 0.942). Male patient improver kidneys had larger volumes than female kidneys. However, there was no significant between sex difference in the PV:isoSK-GFR ratio.

### Control kidneys as the reference group

**Fig 3:**
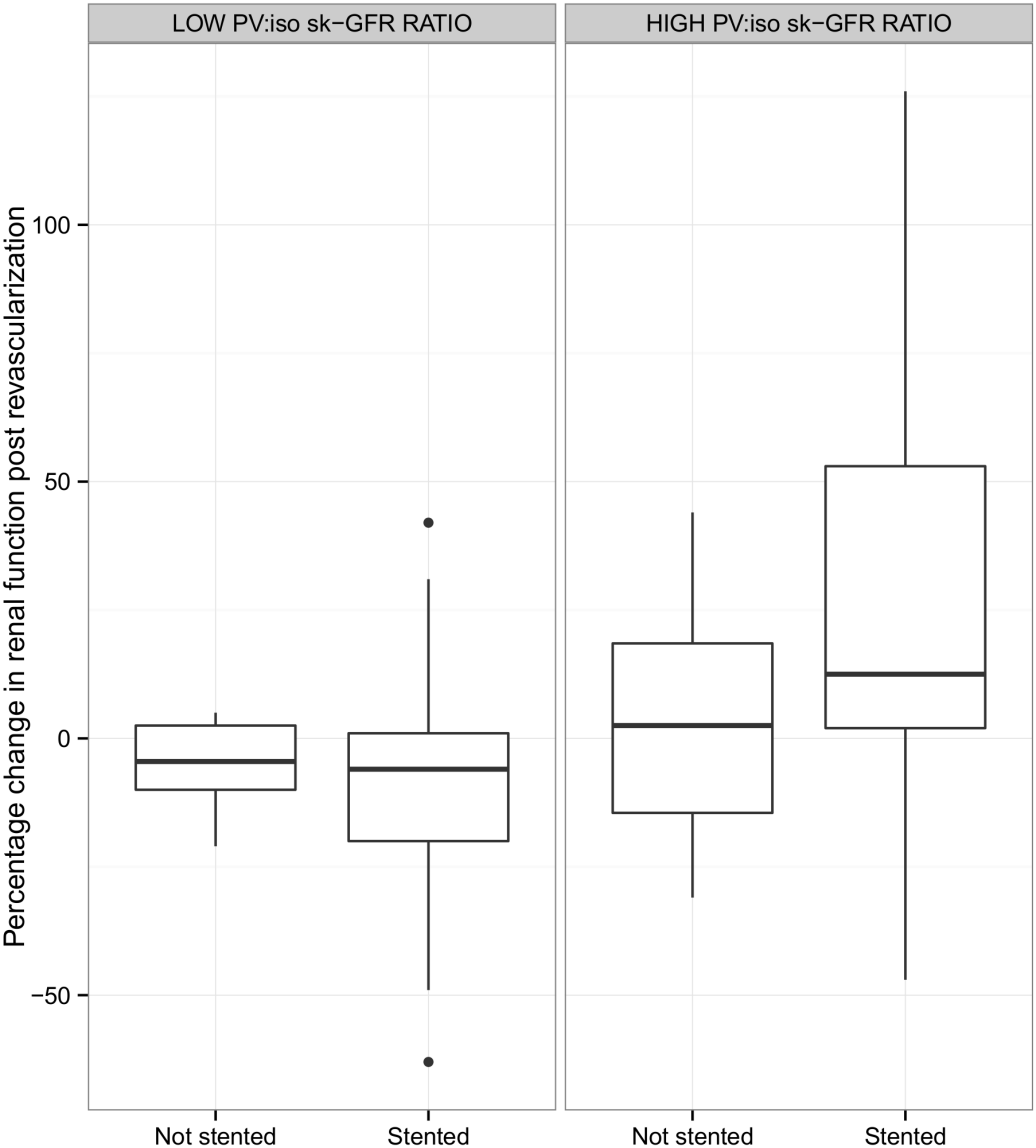
Percentage change in isoSK-GFR at 4 months post-revascularization grouped according to PV:isoSK-GFR ratio. “High ratio” indicates those kidneys which had a PV:isoSK-GFR that exceeded the 95% CI for a given volume of a control kidney.

Control kidneys were grouped into 5 reference categories according to their baseline volumes (ml) and isoSK-GFR. The PV:isoSK-GFR ratios were calculated for each volume group (Table 3). The ratio of PV:isoSK-GFR increased as the renal volume increased. In the smallest volume group (<85mL), the mean PV:isoSK-GFR was 4.5 (0.5–9.5). By comparison, in the largest volume group (>170mL), the mean PV:isoSK-GFR was 10.8 (0.1–21.7). Analysis using these reference groups showed that both stented and contralateral kidneys with baseline PV:isoSK-GFR that exceeded the 95% CI for a given volume of a control kidney were more likely to show renal functional improvement post revascularization (Fig 3).

**Table 3:** PV:isoSK-GFR characteristics of control kidneys used to establish 95% CI.

Volume group (ml)	N	PV:isoSK-GFR (mean (95% CI) min^-1^)
<85.0	3	4.5 (0.5–9.5)
85 to 110	4	5.0 (3.6–8.4)
110.01 to 140	6	5.4 (2.7–8.1)
140.01 to 170	10	5.9 (4.6–7.3)
>170	5	10.8 (0.1–21.7)

### Sensitivity and specificity

**Fig 4:**
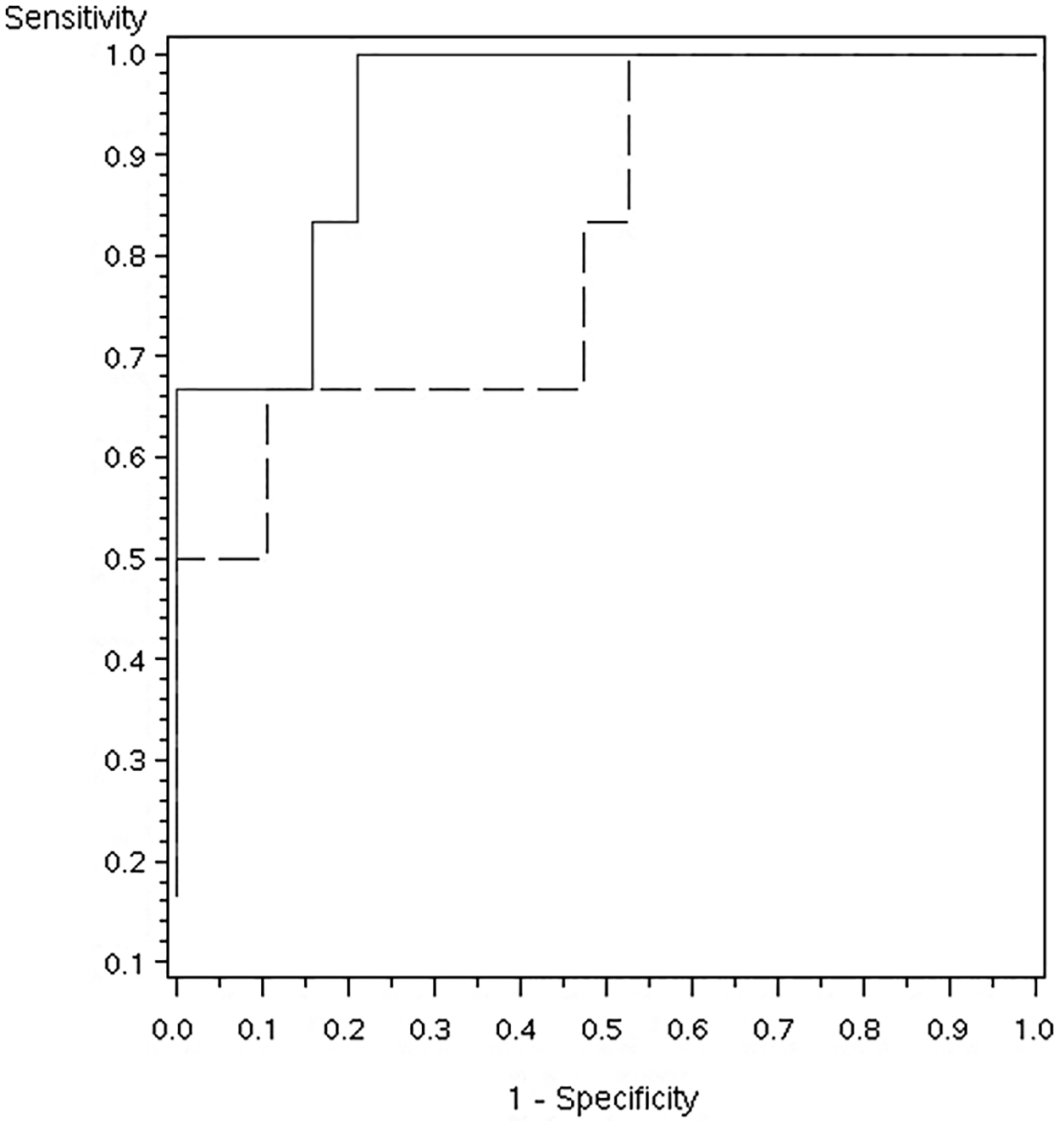
Receiver operating characteristic curves for >15% improvement in isoSK-GFR following revascularization. Dashed line represents isoSK-GFR (AUC 0.81). Solid line represents addition of PV:isoSK-GFR values (AUC 0.93).

The area under the ROC curve (AUC) for baseline isoSK-GFR in predicting a >15% increase in isoSK-GFR following revascularization was 0.81. When PV:isoSK-GFR was added this increased to 0.93 with an integrated discrimination improvement of 0.27, p = 0.001 (Fig 4).

The sensitivity and specificity of PV:isoSK-GFR ratio in predicting beneficial functional outcomes for stented kidneys were 64% and 88%, respectively (Table 4). The ratio was more sensitive but less specific in contralateral kidneys (80% and 67%, respectively). The positive predictive value of the PV:isoSK-GFR ratio for improver response was 75% in stented and 80% in contralateral kidneys. The negative predictive value was 82% in stented and 67% in contralateral kidneys. The area under the curve was 0.938, p <0.0001 for combined stented and contralateral kidneys.

**Table 4:** Comparison of predicted versus actual outcomes for individual stented kidneys.

	Actual response	
Predicted response	Improver (% of predicted)	Non-improver (% of predicted)
Improver	9 (64)	5 (36)
Non-improver	3 (12)	23 (88)

### Survival analysis

**Fig 5:**
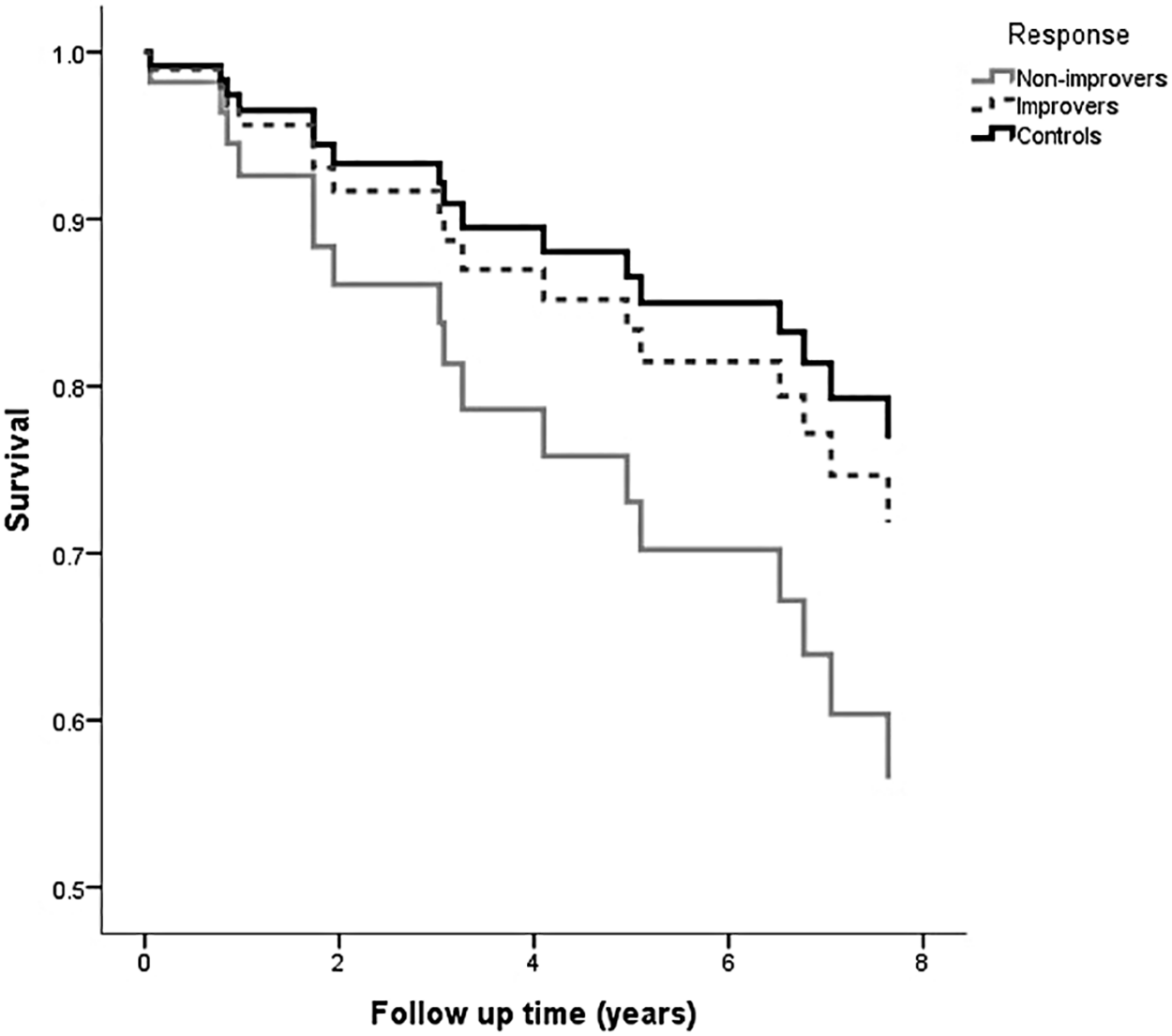
Survival curves for revascularized patients divided into improvers versus non-improvers, and comparing with non ARVD control patients, adjusted for baseline age, co-morbidities, and GFR.

Over a mean 6.1±2.6 years follow up after revascularization, there were 12 deaths in the treatment group (40%); 5 patients progressed to end stage kidney disease necessitating dialysis (28%), of whom 2 died. The estimate of mean survival time for improvers was 6.7 year (95% CI 5.3–8.2 years) compared with 5.7 (4.3–7.1) years in non-improvers, and 7.4 (6.3–8.5) years in the control group. In a Cox proportional hazard model adjusted for baseline age, GFR, gender, diabetes, blood pressure, and coronary artery disease the hazard ratio for death in improvers versus non-improvers was 0.51 (0.16–1.61), p = 0.251. Survival curves comparing improvers with non-improvers and showing control patients is found in Fig 5. There were no statistically significant differences in baseline characteristics between improvers and non-improvers (a full comparison of characteristics is found in online Table 1).

The Cox proportional hazard model was repeated to compare those predicted to respond versus those predicted to not respond, based on isoSK:GFR. Here, the adjusted hazard ratio for death in predicted improvers versus predicted non-improvers was 0.54 (0.15–1.95), p = 0.354.

To observe the potential effect of differences in blood pressure control after revascularization on outcome in improvers versus non-improvers, we compared baseline blood pressure and change in blood pressure at one month after revascularization between the two groups. The mean baseline systolic blood pressure in improvers was 159±25 mmHg compared with 158±33 mmHg in non-improvers (unpaired t-test p = 0.774); baseline diastolic blood pressures were 87±17 mmHg and 78±17 mmHg, respectively (p = 0.143). The mean changes in systolic blood pressure were -19±42mmHg in improvers and -17±63mmHg (p = 0.463) in non-improvers, and change in diastolic blood pressure was -6±18mmHg and -8±32mmHg respectively, (p = 0.865).

## Discussion

The most important finding of this study was that a higher PV:isoSK-GFR ratio pre-revascularization is noted in patients whose renal function improves after revascularization. Stented kidneys which improved had a ratio 3 times higher than those which deteriorated or remained stable. The higher the PV:isoSK-GFR, the more specific the ratio was in determining outcome, although the sensitivity remained moderate (Sig 1). Using ROC analysis, RAS kidneys improved 94% of the time if PV:isoSK-GFR is >95% CI of the control group. In this study, 27% of kidneys improved, which is comparable to other studies that have reported on renal functional response. This emphasizes the need for suitable pre-selection criteria [19].

Importantly, improvement was seen even in kidneys with small SK-GFR (6 revascularized kidneys had baseline isoSK-GFR <10ml/min). These kidneys would not usually have been considered suitable for revascularization by most clinicians. However, a previous twin centre study has shown that the most significant improvements in renal function may actually accompany revascularization of ARVD patients with advanced CKD [7], and the current study suggests that patients could be delayed from progressing to dialysis should the PV:isoSK-GFR ratio of these low function kidneys be suitably large.

Another finding of this study is that some non-ARVD, non-revascularized kidneys contralateral to the revascularized kidney also experienced an improvement in renal function if they had a larger baseline PV:isoSK-GFR. This was more likely in but not exclusive to cases where the stented kidney improved.

We presume that this improvement in contralateral kidneys relates to neurohumoral changes (e.g. changes in renin, and angiotensin) or sympathetic nerve activity, or changes in blood pressure control. Thus even kidneys not directly affected by RAS may have the potential for improvement, and the implication is that the PV:SK-GFR may serve as a surrogate of renal functional reserve in either kidney.

The improver contralateral kidneys with larger PV: isoSK-GFR did not demonstrate a significant drop in this ratio post revascularization, unlike the stented kidneys which did. Both volume and isoSK-GFR improvements occurred in the contralateral kidneys, whereas the isoSK-GFR improvement seemed to exceed that of volume expansion in the stented kidney. One hypothesis to explain these differences could be that ‘hibernating parenchyma’ was present within the improver stented kidneys, with return of blood flow to the ischaemic tissue leading to proportionately greater improvement of GFR rather than volume, given that irreversible structural change had not occurred. In our previous pilot study [12], we found that patients who had high PV:isoSK-GFR kidneys with significant RAS who were treated conservatively were 7 times more likely to show significant decline of eGFR at 1 year compared to their revascularized counterparts. Conversely, with revascularized patients, treatment of a significant RAS kidney that had a high PV:SK-GFR ratio was 28 times more likely to show GFR improvement compared to conservatively managed patients. A large PV:isoSK-GFR is thought to represent the fact that ‘hibernating’ renal tissue is present which is not irreversibly damaged and which is still salveagable with revascularization.

The survival analysis showed a numerical but not statistical survival benefit for improvers over non-improvers. In fact, that outcome for improvers more closely matched that of the control patients than the non-improvers. This must be taken in the context of the fact that this study was not powered to a survival analysis. Nonetheless, such a trend towards improved survival in the improver group supports the hypothesis that not only can PV:isoSK-GFR predict the likelihood of a renal functional response to revascularization but that such a response is a surrogate for improved prognosis for ARVD patients. Given the neutral results of ASTRAL and CORAL indicating that revascularization should not be seen as first line therapy for the majority of patients with ARVD, a simple technique such as that described here may enhance the required screening to select those patients who should be revascularized.

The key limitation of this study is the small patient numbers. This was a pilot study and not designed to provide conclusive answers. Nonetheless, it does inform us that this is an area of potential clinically utility in the future. A second limitation of this study is the two-stop nature of the diagnostic tool. A one-stop approach would benefit patients and likely be more cost-effective in day to day practice. We have previously described the use of BOLD MRI signal in predicting renal functional response to revascularization with similar sensitivity and specificity to the volume based approached described here [20]. The use of MRI to accurately measure SK-GFR is emerging [21]. Alternatively, and for patients who are unable to undergo MRI (e.g. those with heart failure and device in situ), CT-measured volume and GFR may provide an alternative approach.

## Supporting information

S1 Fig (XLSX)
